# 

**Published:** 1895-07-20

**Authors:** 


**Notices of Births, Deaths, and Marriages** are inserted in this column at a charge of 2s. 6d. for an announcement not exceeding four lines.

**Notice to Advertisers and Subscribers.**

All Advertisements, Orders for Copies of The Hospital and Business Communications should be addressed to **The Manager, NOT TO THE EDITOR,** The Hospital, 428, Strand, London, W.C.

The Cost of Subscription, post free, is as follows:—Per week, 2½d.; per quarter, 2s. 8d.; per half-year, 5s. 3d.; per annum, 10s. 6d. Foreign:—Per Annum, 15s. 2d.; payable, in all cases, in advance.

### NOTICE TO CORRESPONDENTS.

All MS., Letters, Books for Review, and other matters intended for the Editor should be addressed The Editor, The Lodge, Porchester Square, London, W.

The Editor cannot undertake to return rejected MS., even when accompanied by stamped directed envelope.

**"Burdett's Hospital Annual," 1895,**
Now Ready.

Sixth year of publication. 950 pp. Scarlet cloth, gilt lettered. Price 5s. A most complete guide to British, Colonial, Indian, and American Hospitals, Dispensaries, Nursing and Convalescent Institutions, and Asylums. A few copies of the "Annual" for 1894 may still be obtained on application to the Publishers, The Scientific Press (Limited), 428, Strand, London, W.C.

**NOTICE.—Vol. XVII. of "The Hospital" (October, 1894, to April, 1895), in handsome cloth binding, is on sale at the Office of this Journal. Price 6s. Cases for binding the Numbers contained in this Volume, 1s. 6d. Index to Vol. XVII., 2½d., post free.**

**The Monthly Part for July is now ready, price 6d.; by post, 8d.**

### SCRAPS AND GLEANINGS.

A great fancy fair, fête, and gala have been held in aid of Barrington's Hospital, Limerick.

The proposed site for the Manchester Smallpox Hospital at Carrington is causing much discussion.

At Rome the principal foundling asylum has been closed because of the great mortality among the patients.

Plans for the erection of the Thanet Joint Hospital Board at an estimated cost of £12,000 have now been prepared for presentation to the Local Government Board.

The customary annual bazaar in aid of the Samaritan and Convalescent funds and free beds of the Norwood Cottage Hospital has now been held. The proceeds of the sale amounted to £120.

The legacy of £2,000 left to the Adelaide Hospital, Dublin, by Mr. Vance, of London, has been expended partly in extending and improving the sanitary arrangements of the surgical wards, &c., and in additions to Victoria House.

For the contemplated extension of the Tynemouth Infirmary it is estimated that £2,000 will be required, and at the completion of the same £200 extra subscriptions per annum will be necessary towards the support of the increased accommodation.

Training schools for children's nurses are, we learn, to be shortly established in connection with the Women's Lyceums in Paris. Such women as go through this training will be presented with a diploma, and become certified nurses for children.

Nearly half the sum already raised in Paris towards the Charcot Memorial has been received from outside sources. Another appeal has been issued to French physicians to further augment the funds of this scheme for immortalising their countryman's name.

An imposing demonstration in aid of the funds of the Montague Cottage Hospital of Mexborough and the Doncaster Infirmary was held the first week of this month at Canisbrough, the organisers of which were the Cadely Main Miners and Glass Blowers' and other friendly societies.

The French Institute has decided to open a subscription list for raising a statue to Lavoisier in his native city of Paris. The committee are anxious that the monument be raised internationally, so as to give all admirers of Lavoisier an opportunity of attesting their recognition to one of the greatest scientists of his day.

The East Suffolk Hospital is still in the condition of so many other charitable institutions of this kind. It is doing more work without any corresponding increase in income. The number of in-patients has increased during the past year by 183, and for the patients, as a whole, better provision has been made than formerly.

The scheme for extending the York Dispensary by adding to it a maternity department was successfully inaugurated lately at a public meeting. The Duke of York, who presided, claimed for the new movement the sympathy of the public on the ground that it is in harmony with all the other charitable agencies in the city.

The annual meeting of the governors and subscribers of the Great Yarmouth Hospital has just taken place. The report presented is a satisfactory one, and the estimation in which the work of this institution is held is reflected in the support it is now receiving. Various extensions are being now seriously contemplated.

A rather remarkable collection for the Hospital Sunday Fund was made round the Christian Evidence stand of the Oxford House in Victoria Park, after Mr. Winnington Ingram had finished his usual lecture in the afternoon, and realized 10 guineas, all taken in coppers. Altogether from the Oxford House agencies £44 has been collected.

The Commission of the Académie des Sciences at Paris has published the report dealing with the feasibility of the project of M. Andrée for attaining the North Pole by means of a balloon. This expresses a negative opinion on the Pole being reached by surface exploration. M. Andrée proposes to start by balloon from Spitzbergen with the hope of reaching this point.

The report of the Retford Dispensary and Cottage Hospital points out that the two specific points upon which stress was laid in previous years, viz., the increase of the nursing staff and the obtaining of new suitable premises, had been accomplished, both of these measures having resulted in a very marked increase in the efficiency and success of the institution.

The Act just introduced by the Lord Chancellor entitled, An Act to Amend the Inebriates Acts, enlarges on the committal of inebriates to a retreat. With regard to this it is proposed that the High Court may, on satisfactory evidence, for any person who is an habitual drunkard, make an order for his committal to such retreat for a term of not less than six months.

A public meeting has just been held at the Axminster Cottage Hospital to consider the financial position of the institution. A circular had been issued to the effect that unless a large support was now forthcoming the hospital must be closed at Michaelmas. Unless there is a guarantee of £130 a year the committee will not be able to carry on the working of the hospital.

Attention is being drawn to the poverty-stricken condition of the Liverpool Hospital for Consumption; to its inconvenience and limited accommodation; as also to its lack of modern appliances and apparatus. A local paper draws an interesting but distressing comparison between this institution and the National Hospital at Ventnor and the Brompton Hospital for Consumption.

The fourteenth annual report of the Sarah Nicol Memorial Cottage Hospital at Llandudno has been presented. The Male Convalescent Home, forming part of this institution, has during the past year done excellent and useful work in providing a home for a greater number of convalescents than in any previous year. After all liabilities of the past year have been discharged a surplus of £143 is shown on the balance-sheets.

The annual meeting of the friends and supporters of the Coombe Lying-in Hospital at Dublin has just taken place. In presenting their report, the directors of this charity state they have every confidence that the record of the work done during the year will prove the efficient condition of the hospital in all its branches. The total cases attended through hospital extern, midwifery, and dispensary branches for the twelve months was 11,568.

### BOOKS RECEIVED.

WALTER SCOTT.

"Sonia Kovalevsky," A. C. Leffler.

CASSELL AND CO., LTD.

"Good Morning—Good Night." By the author of "Beneath the Banner."

"Great Eastern Railway Guide to the Continent." Edited by Percy Lindley.

J. AND A. CHURCHILL.
"Manual of Botany." Reynolds Green.
"Manual of Botany, vol. I." Reynolds Green.

SWAN SONNENSCHEIN.
"Climates of the Geological Past" Eug: Dubois.
"Microbes and Disease Demons." Dr. Berdoe.

JOHN BALE AND SONS.

"Early Scoliosis." Percy G. Lewis.

C. GRIFFIN AND CO.

"Disinfection and Disinfectants." S. Rideal.

**Periodicals and Pamphlets.**—Westminster Review, Strand Magazine, Picture Magazine, Bristol Medico-Chirurgical Review, British Gynecological Journal, Conditions of Radical Cure in Cancer, The Review of the Churches, Birmingham Medical Review.

### THE STUDENT OF MEDICINE.

### APPOINTMENTS.

J. W. Ballantyne, M.D., F.R.C.P.E., F.R.S.Edin., appointed Examiner in Midwifery in the University of Aberdeen. E. C. Bridges, M.B., B.S.Durh., M.R.C.S., L.R.C.P., appointed House Surgeon to the Great Northern Central Hospital. A. Charpentier, M.D.Durh., appointed Outdoor Medical Officer for the Uxbridge District of the Uxbridge Union. C. D. Christmas, M.D.Brux., M.R.C.S., L.R.C.P.Lond., appointed Medical Officer for the Streatham District of the Clapham Union. T. W. H. Garstang, B.A.Oxon, M.R.C.S., appointed Medical Officer of Health to the Knutsford Urban District Council. G. G. Gidley, L.R.C.P. Lond., M.R.C.S., appointed Medical Officer for the Collumpton and Kentisbere District of the Tiverton Union. G. Goodman, L.R.C.P.I., L.R.C.S.I., L.M.R.C.P., L.M., appointed Medical Officer of Health to the Rural Sanitary District of the District Council, Brigg Union. W. H. Goodman, L.D.S.R.C.S.Eng., appointed Surgeon to the Devon and Exeter Dental Hospital. W. C. Greig, M.B. and C.M.Edin., appointed Hon. Physician to H.B.M.'s Legation at Tangier. J. E. Halpin, L.R.C.S.I., L. and L.M.K.Q.C.P.I., appointed Public Vaccinator for Fifth District,

THE STUDENT OF MEDICINE—*continued.*

Mansfield Union. Mr. J. C. Hines, appointed Medical Officer for the Burwash District of the Ticehurst Union. E. Johnstone, M.B., appointed Medical Officer for the Clayton District of the Prestwich Union. Dr. Jones, appointed Medical Officer for the Grangetown District of the Cardiff Union. Dr. Mintner, appointed Medical Officer of the Workhouse and for the Uxbridge District of the Uxbridge Union. Z. B. Mudge, L.R.C.P.Lond., M.R.C.S.Eng., appointed Medical Officer of Health to the Phillack Urban District Council. R. W. Nairn, M.B., C.M.Glasg., appointed Medical Officer for the Farnsfield District of the Southwell Union. W. Permewan, M.D. Lond., F.R.C.S.Eng., appointed Honorary Laryngologist to the Royal Southern Hospital, Liverpool. E. V. Phillips, M.R.C.S.Eng., L.R.C.P.Lond., D.P.H.R.C.S.I., appointed Medical Officer and Public Vaccinator to the No. 5 District, Market Harborough Union; Medical Officer, Market Harborough Rural Sanitary District; and Medical Officer of Health, Oxenden Rural Sanitary District. T. Rhind, M.R.C.S. Eng., L.R.C.P.Lond., appointed Medical Officer and Public Vaccinator No. 1 Western District, Billesdon Union. E. W. Selby, M.B., B.S.Lond., F.R.C.S.Eng., appointed Honorary Surgeon to the Doncaster General Infirmary and Dispensary. J. S. Sewill, M.R.C.S., L.R.C.P., L.D.S., appointed Dental Surgeon to the St. Marylebone General Dispensary, Welbeck Street, W. Dr. T. Smith, appointed Medical Officer for the Bampford Speke District of the St. Thomas Union. C. J. Symonds, M.S., M.D.Lond., F.R.C.S.Eng., appointed Consulting Surgeon to the Stockwell Orphanage. F. J. de Coverly Veale, M.B., Ch.B.Vict., appointed Assistant Medical Officer at the County Asylum Lancaster.

### VACANCIES.

The following vacancies are announced this week, the amount of salary in each case being given after the name of the institution:

### Resident Medical Officers.

Borough of West Ham.—Medical Superintendent for the Borough Hospital for Infectious Diseases at Plaistow. Salary £200 per annum, with annual increment of £10 up to £250, with apartments, rations, and washing. Not less than 25 years of age. Applications, on forms provided, to be sent to F. E. Hilleary, Town Clerk, Town Hall, West Ham, E., by July 23rd.

Chichester Infirmary.—House Surgeon. Salary £80 per annum, with board, lodging, and washing. Applications to the Secretary by August 12th.

Coventry and Warwickshire Hospital.—Resident Assistant House Surgeon. Appointment for six months. Salary £15, board (exclusive of beer, wines, and spirits), residence, washing, and attendance. Applications to the Secretary by July 20th.

Great Northern Central Hospital, Holloway Road, N.—Junior House Surgeon. Appointment for six months. Board, lodging, and laundry provided. Applications, on forms provided, to be sent to Lewis H. Glenton Kerr, Secretary, by July 29th.

National Sanatorium for Consumption and Diseases of the Chest, Bournemouth.—Resident Medical Officer. Salary £80 per annum, with board, lodging, and washing. Applications to the Secretary by July 25th.

Salford Union Infirmary, Hope, near Eccles.—Assistant Medical Officer; doubly qualified. Salary £130 per annum, with furnished apartments in the infirmary. Applications, endorsed "Assistant Medical Officer," to T. H. Bagshaw, Clerk to the Guardians, Union Offices, Eccles New Road, Salford, by July 23rd.

Sheffield General Infirmary.—House Surgeon and Senior Assistant House Surgeon, doubly qualified. Salary for the former, £120 per annum, with a prospective advance of £10 per year for the second and third years; and for the latter £80 per annum, with board, lodging, and washing. Applications to the "Medical Staff of the Sheffield General Infirmary, to the care of the Secretary," by July 13th. The election will take place on July 26th.

York Dispensary.—Resident Obstetric House Surgeon; unmarried. Salary £150 per annum, with furnished apartments, coals, and gas. Applications to Mr. W. Draper, De Grey House, York, by July 23rd.

### Non-Resident Medical Officers.

Bradford Infirmary and Dispensary.—Honorary Physician. Applications to the Secretary, by July 22nd.

British Institute of Preventive Medicine.—Assistant Bacteriologist in the Anti-toxin Department. Salary £150 a year. Applications to the Director, by July 22nd.

Guy's Hospital Medical School.—Lecturer on Public Health. Applications to the Treasurer, Counting House, Guy's Hospital, S.E., by July 20th.

Hospital for Women, Soho Square.—Non-Resident House Physician. Appointment for three months. Applications to the Secretary by July 31st.

University of Glasgow.—Two Examiners for Degrees in Medicine to Examine in Chemistry and Materia Medica respectively. Applications to Alan E. Clapperton, Secretary of the Glasgow University Court, 91, West Regent Street, Glasgow, by July 25th.

MEDICAL SUPERINTENDENT.

